# Breast arterial calcifications on mammography and risk of stroke: a systematic review and meta-analysis

**DOI:** 10.3389/fneur.2026.1716747

**Published:** 2026-02-05

**Authors:** Sherief Ghozy, Seyed Behnam Jazayeri, Mohamed Ahmed Ali, Muhayman Sadiq, Pedram Pakzamir, Mohammad Reza Fattahi, Abdolkarim Haji Ghadery, Rachana R. Borkar, Adam A. Dmytriw, Ramanathan Kadirvel, Amy Lynn Conners, David F. Kallmes

**Affiliations:** 1 Department of Radiology, Mayo Clinic, Rochester, MN, United States; 2 Department of Neurologic Surgery, Mayo Clinic, Rochester, MN, United States; 3 Comprehensive Stroke Center, Department of Neurosciences, University of California San Diego, San Diego, CA, United States; 4 Qena Faculty of Medicine, Qena University, Qena, Egypt; 5 Department of Diagnostic and Interventional Imaging, McGovern Medical School, UTHealth Houston, Houston, TX, United States; 6 Faculty of Dentistry, Islamic Azad University, Borujerd, Iran; 7 School of Advanced Technologies in Medicine, Shahid Beheshti University of Medical Sciences, Tehran, Iran; 8 Department of Radiology, Advanced Diagnostic and Interventional Radiology Research Center (ADIR), Tehran University of Medical Sciences, Tehran, Iran; 9 Department of Radiology and Biomedical Imaging, Yale University School of Medicine, New Haven, CT, United States; 10 Neuroendovascular Program, Massachusetts General Hospital & Brigham and Women's Hospital, Harvard Medical School, Boston, MA, United States; 11 Nuffield Department of Surgical Sciences, Medical Sciences Division, University of Oxford, Oxford, United Kingdom; 12 Neurointerventional & Neuroanalytics Consortium (NAN-C), School of Medicine, Toronto Metropolitan University, Toronto, ON, Canada

**Keywords:** breast arterial calcification (BAC), cerebrovascular disease, mammography, meta-analysis, stroke

## Abstract

**Background:**

Breast arterial calcifications (BAC) are commonly observed as an incidental finding on screening mammography and have been linked to cardiovascular disease. Whether BAC is independently associated with stroke risk remains uncertain.

**Methods:**

We conducted a systematic review and meta-analysis in accordance with PRISMA 2020 guidelines. PubMed, Embase, and Scopus were searched from inception to May 2024 for cohort studies evaluating the association between BAC and stroke. Eligible studies included women undergoing mammography with documented BAC status and subsequent stroke outcomes. Data on patient characteristics and vascular risk factors were extracted. Study quality was appraised using the Joanna Briggs Institute (JBI) Critical Appraisal Checklist. Random-effects meta-analyses with restricted maximum likelihood (REML) estimation were performed to pool risk ratios (RRs) and mean differences (MDs). Heterogeneity was assessed with the I^2^ statistic, and publication bias with Egger’s test.

**Results:**

Ten cohort studies including 52,413 women were analyzed. The presence of BAC was associated with more than a twofold increased risk of stroke (RR 2.09, 95% CI 1.58–2.75; I^2^ = 64.4%). This association persisted after adjustment for age, diabetes, hyperlipidemia, and menopausal status. Compared with BAC-negative women, those with BAC were significantly older (MD 7.07 years, 95% CI 5.44–8.70) and more frequently hypertensive (RR 1.48, 95% CI 1.23–1.78), diabetic (RR 1.67, 95% CI 1.38–2.02), hyperlipidemic (RR 1.27, 95% CI 1.07–1.51), and postmenopausal (RR 1.26, 95% CI 1.00–1.59). Interestingly, BAC was less common among smokers (RR 0.62, 95% CI 0.45–0.86). Egger’s test showed no evidence of publication bias (*p* = 0.143).

**Conclusion:**

BAC detected on screening mammography is independently associated with an increased risk of stroke, even after accounting for traditional risk factors. These findings support BAC as a promising, underutilized imaging biomarker of cerebrovascular risk in women and highlight the need for standardized reporting and prospective validation.

## Introduction

Breast arterial calcifications (BAC) are a common incidental finding on screening mammography, traditionally regarded as benign and not routinely reported (1). Emerging evidence, however, suggests that BAC reflect systemic vascular pathology and are associated with coronary artery disease (CAD) and adverse cardiovascular disease (CVD) outcomes (2–4). Recognition of sex-specific risk factors for CVD has been limited, with traditional risk models often overlooking conditions unique to women such as hypertensive disorders of pregnancy, gestational diabetes, and menopause (5, 6). Developing female-specific screening and risk stratification strategies is therefore essential. Mammography, already widely implemented for breast cancer detection, offers a unique opportunity to also capture cardiovascular risk markers in women. The American College of Radiology (ACR) recommends routine mammographic screening beginning at age 40 through 74, with annual screening at least until 55 (7), providing a broad population-level platform for opportunistic vascular risk assessment.

Stroke, defined as an acute focal neurological deficit due to vascular injury (ischemic or hemorrhagic), is the second leading cause of death and disability worldwide (8). Its burden is projected to rise, particularly among women, in whom traditional cardiovascular risk models often underestimate risk. Current tools such as the Framingham Stroke Risk Profile do not incorporate specific imaging biomarkers like BAC, which may represent an underutilized predictor of cerebrovascular events (9).

Pathologically, BAC represent medial arterial calcification (Mönckeberg sclerosis), distinct from intimal atherosclerotic plaques. Medial calcification contributes to arterial stiffness and hemodynamic changes that predispose to end-organ damage rather than luminal obstruction (10). The prevalence of BAC is reported in 12–30% of screened women and increases with age, parity, diabetes, and chronic kidney disease (11). Unlike atherosclerotic calcifications, BAC appear more strongly linked to mineral metabolism and diabetes, and less to smoking or dyslipidemia, supporting its role as an independent vascular risk marker.

Although several studies and meta-analyses have reported associations between BAC and broad CVD outcomes, including CAD, heart failure, and peripheral vascular disease, stroke has been comparatively understudied (1, 2, 12). Findings from available studies are inconsistent, leaving uncertainty about the role of BAC in stroke prediction. Yet, stroke is of particular interest, as cerebral small-vessel disease and large-artery stiffness may share pathophysiological pathways with medial calcification (13). Furthermore, multiple studies have reported independent associations between BAC and mortality and CVD events in women aged 40–59, the demographic most frequently undergoing mammographic screening (2, 14).

To date, no systematic review or meta-analysis has examined BAC in relation to stroke as a primary outcome. Prior reviews have pooled heterogeneous cardiovascular events, diluting stroke-specific associations. Our study addresses this gap by synthesizing available evidence on BAC and incident stroke (ischemic and hemorrhagic), while also evaluating patient characteristics and vascular risk factors to clarify the potential role of BAC as a cerebrovascular risk marker.

## Methods

### Search strategy

This review was conducted in accordance with the PRISMA 2020 guidelines. A comprehensive search of PubMed, Embase, and Scopus was performed from inception through February 2025 using terms related to breast arterial calcification (BAC), stroke, and cerebrovascular outcomes. No language or publication year restrictions were applied.

### Eligibility criteria

Studies were eligible if they were prospective or retrospective cohorts that included women undergoing screening or diagnostic mammography, reported the presence of BAC, included a comparator group without BAC, and assessed incident stroke (ischemic or hemorrhagic). Studies reporting only composite cardiovascular outcomes were included only if stroke data could be extracted separately. Reviews, editorials, case reports, and conference abstracts were excluded.

### Study selection

**Figure 1:**
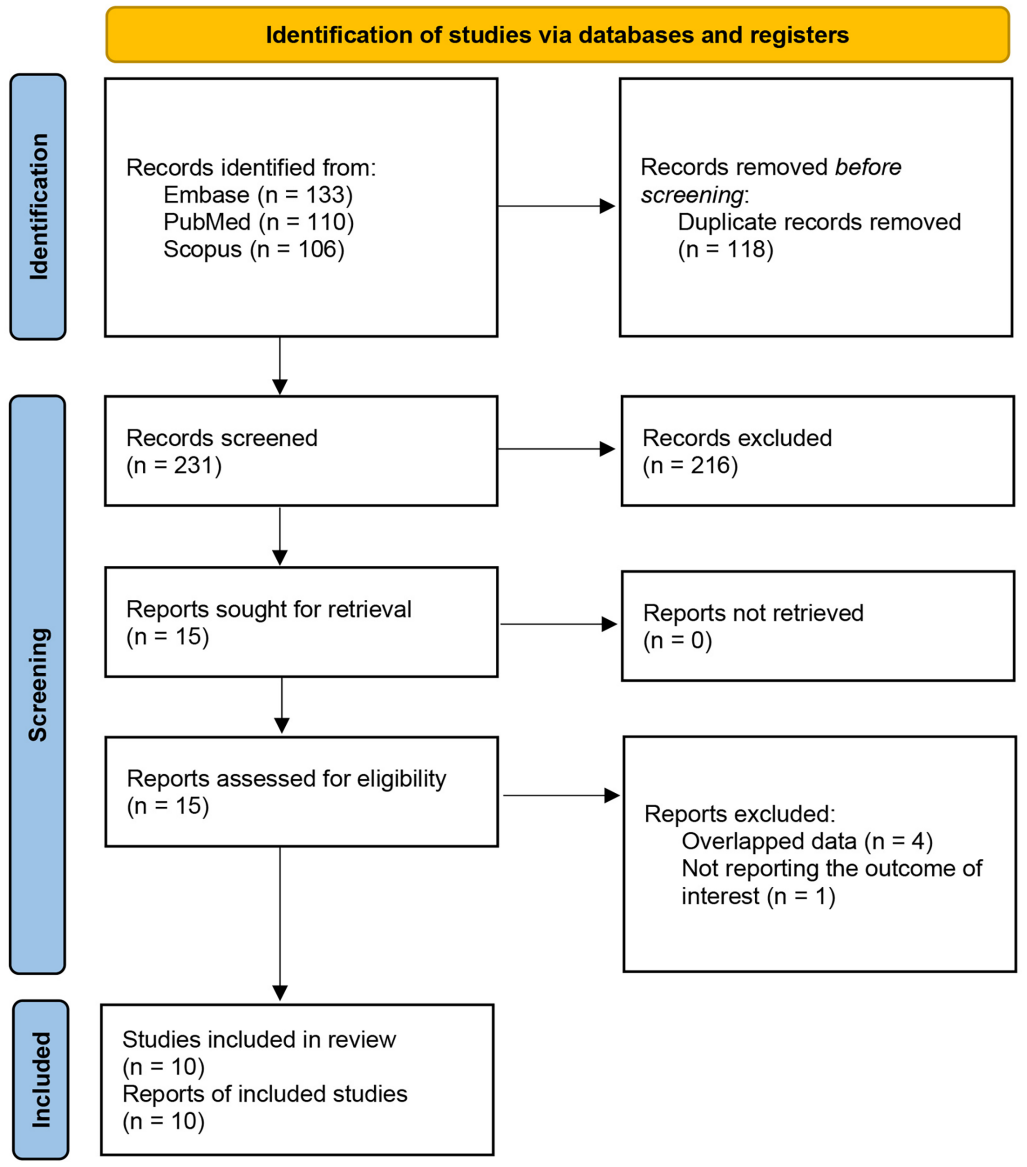
PRISMA flow diagram of search and screening.

Two reviewers independently screened titles and abstracts, and full texts were retrieved for all potentially eligible records. Discrepancies were resolved through discussion and consensus. The process of study identification, screening, and inclusion is summarized in the PRISMA flow diagram (Figure 1).

### Data extraction

Data extraction was performed independently by two reviewers using a standardized template. Extracted data included study design, sample size, characteristics of BAC-positive and BAC-negative groups, methods of BAC ascertainment, stroke outcomes, follow-up duration, and covariates used for adjustment. Additional information on patient comorbidities and risk factors, such as hypertension, diabetes, hyperlipidemia, smoking, and menopausal status—was also collected.

### Risk of bias assessment

The methodological quality of included studies was assessed using the Joanna Briggs Institute (JBI) Critical Appraisal Checklist for Cohort Studies, which evaluates comparability of groups, validity of exposure and outcome measurement, adequacy of follow-up, and appropriateness of statistical analyses.

### Outcomes

The primary outcome was incident stroke, including both ischemic and hemorrhagic events. Secondary outcomes included differences in baseline characteristics between BAC-positive and BAC-negative groups, providing insight into risk factor distributions.

### Data synthesis and statistical analysis

Meta-analyses were conducted in R (version 4.3.2) using the meta and metafor packages. For all outcomes, we performed random-effects meta-analyses using restricted maximum likelihood (REML). Pooled effect sizes are reported as risk ratios (RRs) for dichotomous outcomes and mean differences (MDs) for continuous outcomes, each with 95% confidence intervals (CIs). Between-study heterogeneity was assessed using the I^2^ statistic and Cochran’s Q test, with I^2^ ≥ 50% or *p*-value <0.05 considered evidence of significant heterogeneity. Sensitivity analyses included leave-one-out analyses and influence diagnostics. Publication bias was assessed using funnel plots and Egger’s regression test whenever more than 10 studies were included in the analysis.

## Results

### Study selection

Our initial search across PubMed, Embase, and Scopus yielded a total of 349 records. After removing 118 duplicates, 231 unique records were screened. Of these, 216 were excluded, including conference abstracts (*n* = 15), studies not addressing stroke risk (*n* = 14), irrelevant records (*n* = 178), reviews (*n* = 8), and one withdrawn study. Fifteen full-text reports were retrieved and assessed for eligibility. Following review, five reports were excluded due to overlapping data (*n* = 4) or lack of outcome reporting (*n* = 1). Ultimately, 10 studies met the inclusion criteria and were incorporated into the systematic review and meta-analysis (Figure 1).

### Summary of the included studies

Ten cohort studies (1998–2024) comprising 52,413 women (BAC+ = 7,787; BAC– = 44,626) were included. Per-study totals ranged from 197 to 17,914 participants (BAC + group sizes 70–4,138; BAC– group sizes 125–13,776). Composite endpoints varied across cohorts (e.g., MI + stroke + CVD death; broader 5- to 9-component sets), and outcomes were typically ascertained via medical records or registry linkage with variable follow-up durations (Supplementary Table S1).

### Baseline characteristics of BAC(+) and BAC(−) groups

**Figure 2:**
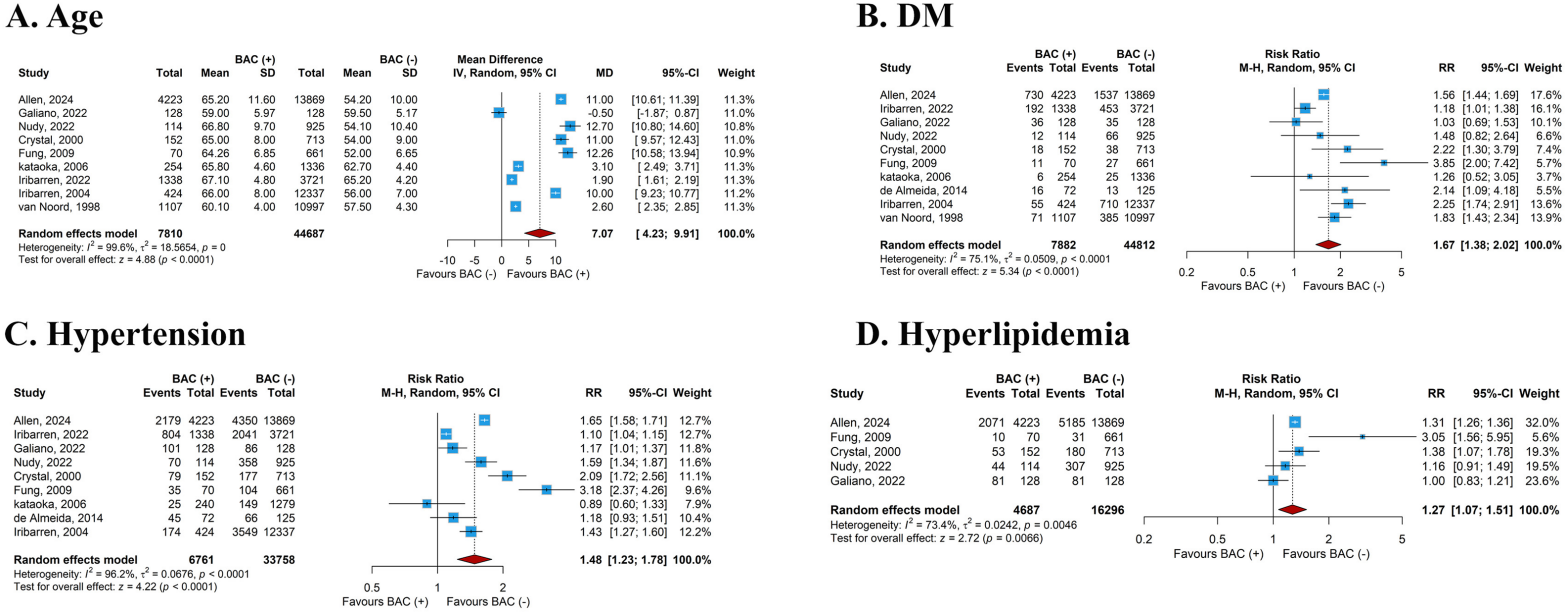
Correlates of BAC presence (baseline comparisons BAC+ vs BAC–).

Patients baseline characteristics are summarized in Supplementary Tables S2, S3. When comparing baseline characteristics between women with and without BAC, several consistent patterns emerged across studies. Women with BAC were significantly older at the time of screening, with a pooled mean difference of just over 7 years (MD 7.07, 95% CI 4.23–9.91). In addition to age, cardiometabolic risk factors were more prevalent in the BAC-positive group. The likelihood of having diabetes mellitus was nearly twofold higher (RR 1.67, 95% CI 1.38–2.02), and hypertension was also more common (RR 1.48, 95% CI 1.23–1.78) (Figure 2). Similarly, the prevalence of hyperlipidemia was modestly elevated (RR 1.27, 95% CI 1.07–1.51).

Reproductive status also differed between the groups. Women with BAC were more likely to be postmenopausal at the time of mammographic screening (RR 1.26, 95% CI 1.00–1.59) (Supplementary Figure S1). Smoking showed an opposite pattern: the prevalence of current smoking was substantially lower among women with BAC compared to those without (RR 0.62, 95% CI 0.45–0.86) (Supplementary Figure S2).

Other clinical characteristics, including the use of antihypertensive or lipid-lowering medications (statins), total cholesterol levels, history of cardiovascular disease, and current use of hormone therapy did not show consistent differences between groups. These findings suggest that while women with BAC share a higher burden of traditional vascular risk factors, some behaviors, such as smoking, may be less common in this population (Supplementary Figures S3–S7).

### Meta-analysis of stroke outcomes

**Figure 3:**
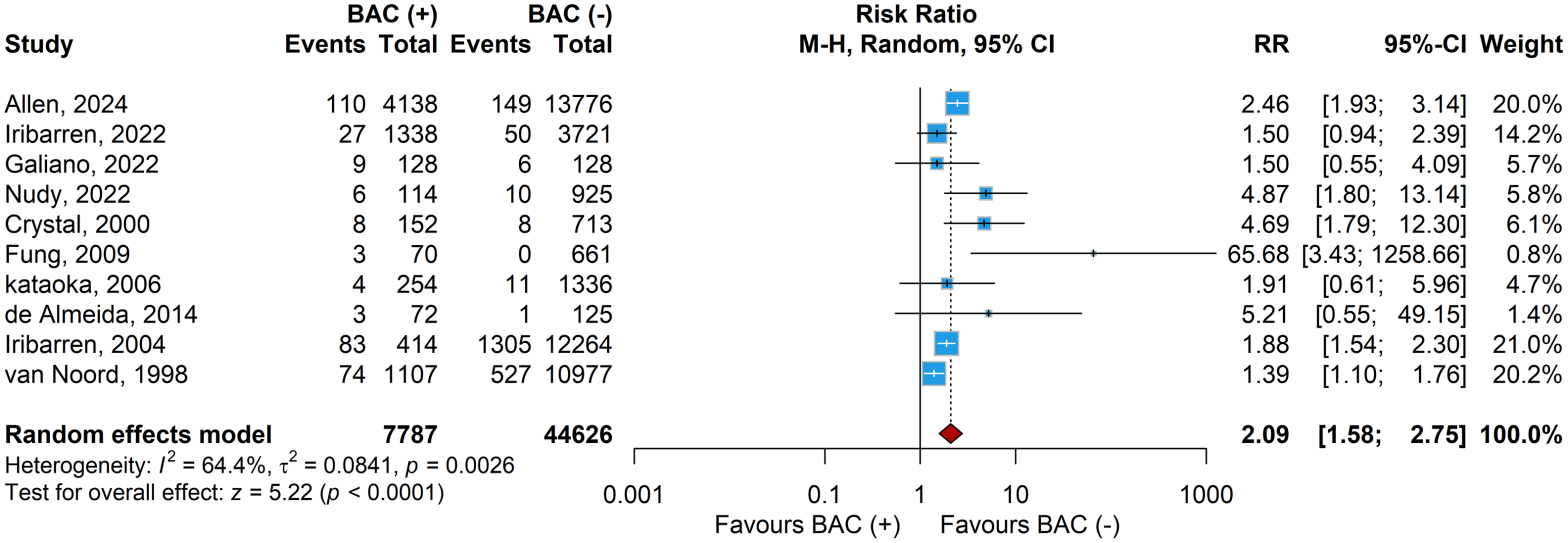
Primary pooled analysis (BAC+ vs BAC–).

A total of 10 studies (*n* = 52,413 women; 7,787 with BAC and 44,626 without BAC) reported on the association between breast arterial calcifications and incident stroke. The pooled analysis using a random-effects model demonstrated that women with BAC had a significantly higher risk of stroke compared with those without BAC (RR 2.09, 95% CI 1.58–2.75, *p* < 0.001; Figure 3). Between-study heterogeneity was moderate (I^2^ = 64.4%, τ^2^ = 0.0841, p for Cochran’s Q = 0.0026).

### Publication bias

Visual inspection of the funnel plot did not suggest substantial asymmetry, and Egger’s regression test was not statistically significant (*p* = 0.143), indicating no evidence of small-study effects (Supplementary Figure S8).

### Sensitivity and influence analyses

Leave-one-out analyses demonstrated robust findings, with pooled risk ratios ranging from 1.97 to 2.29 (all statistically significant), and heterogeneity ranging between 52 and 68% depending on which study was excluded (Supplementary Figure S9). Influence diagnostics (Cook’s distance, DFFITS, covariance ratios, and studentized residuals) did not identify any single study that disproportionately influenced the overall effect (Supplementary Figure S10).

### Quality assessment

Using the JBI Critical Appraisal Checklist for Cohort Studies, all 10 included studies were judged suitable for synthesis. Most cohorts recruited comparable groups, applied consistent and valid methods for exposure assessment, and used appropriate statistical analyses. The main methodological limitations were inconsistent reporting of confounder adjustment and follow-up procedures, although outcome measurement was generally reliable. A detailed breakdown of item-level ratings is provided in Supplementary Figures S11, S12.

## Discussion

In this systematic review and meta-analysis of 10 cohort studies including 52,413 women, the presence of BAC was associated with more than a twofold increased risk of stroke. This association persisted across sensitivity analyses adjusted for age, diabetes, menopause, and hyperlipidemia, with pooled estimates ranging from 1.97 to 2.29 despite moderate heterogeneity. Women with BAC were consistently older (mean difference 7 years) and more likely to have hypertension, diabetes, hyperlipidemia, and postmenopausal status, underscoring BAC as a marker of elevated vascular risk detectable on screening mammography.

A notable finding was the inverse association between BAC and smoking, consistent with prior cohorts and meta-analyses (12, 15–20). Although counterintuitive, this reflects the distinct biology of medial arterial calcification (Mönckeberg sclerosis), which is characterized by osteogenic transformation of vascular smooth muscle cells, matrix vesicle release, and calcium phosphate deposition (21–27). Unlike intimal atherosclerosis, strongly linked to smoking, medial calcification is more closely associated with age, diabetes, and disorders of mineral metabolism. These findings support BAC as a marker of medial arterial disease, a process that increases arterial stiffness and pulse pressure, both established contributors to stroke pathogenesis (28, 29).

The association between BAC and postmenopausal status likely reflects the combined effect of age and hormonal decline. Estrogen supports vascular homeostasis through favorable effects on lipid metabolism and nitric oxide–mediated vasodilation, while its loss promotes oxidative stress, endothelial dysfunction, and vascular calcification (30–34). Because most included studies did not report menopausal age or duration, we were unable to distinguish the impact of hormonal factors from chronological aging. Prospective datasets with detailed reproductive histories are needed to clarify this relationship.

Most studies classified BAC as a binary variable, limiting dose–response analyses. Only Allen et al. (14) applied a structured scoring system and found that greater BAC burden predicted higher mortality, particularly among younger women. Current reporting practices are inconsistent: most radiologists record BAC as present/absent, fewer provide semiquantitative grading, and only 1% use standardized scoring (35). Establishing a consensus reporting system, potentially through Delphi methods, will be critical to determine whether BAC should be incorporated qualitatively or quantitatively into mammography reports. In parallel, AI-based methods are being actively developed to support this transition: for instance, Saccenti et al. (36) validated a deep-learning algorithm that assigns a BAC AI score (0–10), showed strong correlation with radiologists’ manual scoring (r = 0.83), and suggested that this automated quantification might facilitate integration of BAC into routine mammography reporting.

Our findings also highlight the need for clinical guidance. At present, BAC is variably reported, and there is no consensus on whether its detection should prompt further cardiovascular or cerebrovascular evaluation. While residual confounding cannot be excluded, our results suggest that BAC is an independent risk marker for stroke. Given that mammographic screening begins around age 40 for average risk women, well before the average age of first stroke in women (73 years in the United States), identifying BAC could provide an early window for preventive counseling and more aggressive management of modifiable risk factors (37).

This study has several strengths, including its large pooled sample, stroke-specific focus, and risk factor comparisons between BAC-positive and negative women. Limitations include reliance on observational designs, variable covariate definitions, and the inability to differentiate ischemic from hemorrhagic stroke in most studies. Furthermore, menopausal details were inconsistently reported, and the absence of harmonized BAC scoring precluded dose–response analyses.

Future research should prioritize prospective cohorts with standardized BAC scoring systems, stroke subtype–specific analyses, and mechanistic studies to clarify the vascular pathways linking BAC to cerebrovascular risk. Delphi consensus initiatives are needed to establish uniform reporting practices, and interventional studies should test whether incorporating BAC into risk assessment improves stroke prevention strategies. Finally, given the higher prevalence of BAC in Hispanic and Black women, longitudinal studies are warranted to determine how racial and ethnic disparities in BAC translate into differential stroke risk (38, 39).

## Conclusion

BAC identified on screening mammography is strongly associated with incident stroke, independent of traditional vascular risk factors. These findings support BAC as a promising but underutilized biomarker of cerebrovascular risk in women and highlight the need for standardized reporting and prospective validation.

## Data Availability

The data analyzed in this study is subject to the following licenses/restrictions: all data extracted and analyzed for this systematic review and meta-analysis are derived from published studies cited in the manuscript. The datasets generated during the synthesis are available from the corresponding author on reasonable request. Requests to access these datasets should be directed to Sherief Ghozy, Ghozy.Sherief@mayo.edu.
