# Supplementary material for: Breast arterial calcifications on mammography and risk of stroke: a systematic review and meta-analysis
Source: Front Neurol. 2026 Feb 5;17:1716747. doi: 10.3389/fneur.2026.1716747 (PMC12917896; doi:10.3389/fneur.2026.1716747)

**Table S1. Summary of the included studies**

| Study, year | Sample size (n) | | Stroke | | TIA | | Hemorrhagic stroke | | Mortality | | MI | | Heart Failure | | Composite outcome | | Definition of Composite outcome |
| --- | --- | --- | --- | --- | --- | --- | --- | --- | --- | --- | --- | --- | --- | --- | --- | --- | --- |
|  | **BAC+** | **BAC-** | **BAC+** | **BAC-** | **BAC+** | **BAC-** | **BAC+** | **BAC-** | **BAC+** | **BAC-** | **BAC+** | **BAC-** | **BAC+** | **BAC-** | **BAC+** | **BAC-** |  |
| Allen, 2024 | 4138 | 13776 | 110 | 149 | - | - | - | - | 329 (n=4223) | 313 (n=13869) | 36 (n=4204) | 47 (n=13847) | 154 (n=4119) | 144 (n=13792) | 500 (n=4031) | 582 (n=13689) | MI, heart failure, stroke and mortality |
| de Almeida, 2014 | 72 | 125 | 3 | 1 | - | - | - | - | - | - | - | - | - | - | - | - | - |
| Iribarren, 2022 | 1338 | 3721 | 27 | 50 | - | - | - | - | 18 | 15 | 18 | 33 | 33/1331 | 66/3704 | 60 | 95 | MI, Ischemic stroke, CVD death |
| Iribarren, 2004 | 414 | 12264 | 83 | 1305 | 11 | 213 | 12 | 272 | - | - | - | - | 68/424 | 1040/12332 |  |  |  |
| kataoka, 2006 | 254 | 1336 | 4 | 11 | - | - | - | - | - | - |  |  | - | - | 12 | 25 | Coronary heart disease plus stroke. |
| Nudy, 2022 | 114 | 925 | 6 | 10 | - | - |  |  | - | - | 1 | 8 | - | - | 13 | 25 | Any of 5: Angina, Myocardial infarction, Abnormal coronary angiography, Coronary artery bypass graft, Stroke |
| Galiano, 2022 | 128 | 128 | 9 | 6 | - | - | 2 | 2 | - | - | 26 | 8 | 33 | 16 | 232 | 102 | Any of 9: IHD, hypertensive heart disease, valvular heart disease, CHF, Afib, PVD, Ischemic stroke, hemorrhagic stroke, lacunar infarction |
| Crystal, 2000 | 152 | 713 | 8 | 8 | - | - | - | - | - | - | 13 | 24 | - | - | - | - | - |
| Fung, 2009 | 70 | 661 | 3 | 0 |  |  |  |  |  |  |  |  |  | - |  |  | - |
| van Noord, 1998 | 1107 | 10977 | RR: 1.4 (1.1-1.8) for TIA/stroke  total events=577, BAC+: 6.7% and BAC-: 4.8%  -  - | | | | - | - | - | - | 8.50% | 4.30% | - | - | - | - | - |

**BAC = Breast arterial calcification; TIA = Transient ischemic attack; MI = Myocardial infarction; CVD = Cardiovascular disease; IHD = Ischemic heart disease; CHF = Congestive heart failure; Afib = Atrial fibrillation; PVD = Peripheral vascular disease; RR = Risk ratio.**

**Table S2. Summary of patients’ age and medication use**

| Study, year | Age (mean+SD) | | Antihypertensive use | | Statin use | | Current use of hormone therapy | | Past use of hormone therapy | |
| --- | --- | --- | --- | --- | --- | --- | --- | --- | --- | --- |
|  | **BAC+** | **BAC-** | **BAC+** | **BAC-** | **BAC+** | **BAC-** | **BAC+** | **BAC-** | **BAC+** | **BAC-** |
| Allen, 2024 | 65.2+11.6 | 54.2+10.0 | 1313 | 2185 | 1430 | 2517 | - | - | - | - |
| de Almeida, 2014 | 65.6 | 60.7 | - | - | - | - |  | - | - | - |
| Iribarren, 2022 | 67.1+4.8 | 65.2+4.2 | 585 | 1410 | 1003 | 2705 | 128 | 425 | - | - |
| Iribarren, 2004 | 66+8 | 56+7 | - | - | - | - | 58 | 3,431 | - | - |
| kataoka, 2006 | 65.8± 4.6 | 62.7± 4.4 | - | - | - | - | - | - | - | - |
| Nudy, 2022 | 66.8 ± 9.7 | 54.1 ± 10.4 | - | - | - | - | 4 | 59 | 26 | 189 |
| Galiano, 2022 | Mean: 59 years (44–74) | Mean: 59.5 years (44–70) | - | - | - | - | - | - | - | - |
| Crystal, 2000 | 65±8 | 54±9 | - | - | - | - | - | - | - | - |
| Fung, 2009 | 64.26±6.85 | 52.00±6.65 | - | - | - | - | 19 | 70 | - | - |
| van Noord, 1996 | 60.1± 4.0 | 57.5± 4.3 | - | - | - | - | - | - | - | - |

**BAC = Breast arterial calcification**

**Table S3. Summary of patients’ comorbidities**

| Study, year | HTN | | DM | | Total cholesterol | | HLP | | Smoking | | History of CKD | | Menopause | | History of breast cancer | | History of CVD | |
| --- | --- | --- | --- | --- | --- | --- | --- | --- | --- | --- | --- | --- | --- | --- | --- | --- | --- | --- |
|  | BAC+ | BAC- | BAC+ | BAC- | BAC+ | BAC- | BAC+ | BAC- | BAC+ | BAC- | BAC+ | BAC- | BAC+ | BAC- | BAC+ | BAC- | BAC+ | BAC- |
| Allen, 2024 | 2179 | 4350 | 730 | 1537 | 194 (53) | 199 (51) | 2071 | 5185 | 134 | 700 | 358 | 444 | - | - | - | - | 424 | 450 |
| de Almeida, 2014 | 45 | 66 | 16 | 13 | - | - | - | - | 5 | 25 | - | - | All (100%) | | - | - | - | - |
| Iribarren, 2022 | 804 | 2041 | 192 | 453 | 207±39 | 207±37 | - | - | Current: 49 Former: 455 | Current: 149 Former: 1234 | - | - | - | - | - | - | - | - |
| Iribarren, 2004 | 174 | 3549 | 55 | 710 | 6.8±1.3 | 6.5±1.3 | - | - | Current: 3457 Former: 40 | Current: 3457 Former: 1819 | - | - | 342/348 | 8459/10348 | - | - | - | - |
| kataoka, 2006 | 25/240 | 149/1279 | 6/254 | 25/1336 | 6.58±1.19 | 6.52±1.12 | - | - | Current:9/253 former: 72/253 | Current: 123/1322 Former: 406/1322 | - | - | All (100%) | | - | - | 12 | 25 |
| Nudy, 2022 | 70 | 358 | 12 | 66 | - | - | 44 | 307 | 3 | 48 | - | - | 111 | 798 | 9 | 32 | - | - |
| Galiano, 2022 | 101 | 86 | 36 | 35 | - | - | 81 | 81 | - | - | - | - | - | - | - | - | - | - |
| Crystal, 2000 | 79 | 177 | 18 | 38 | - | - | 53 | 180 | 14 | 212 | - | - | 138 | 484 | - | - | - | - |
| Fung, 2009 | 35 | 104 | 11 | 27 | - | - | 10 | 31 | 4 | 10 | - | - | 67 | 277 | - | - | - | - |
| van Noord, 1996 | - | - | 6.40% | 3.50% | - | - | - | - | 14.20% | 28.50% | - | - | - | - | - | - | - | - |

**HTN = Hypertension; DM = Diabetes mellitus; HLP = Hyperlipidemia; CKD = Chronic kidney disease; CVD = Cardiovascular disease; BAC = Breast arterial calcification**

**Figure S1. Pooled risk ratio for postmenopausal status in BAC-positive versus BAC-negative women.**


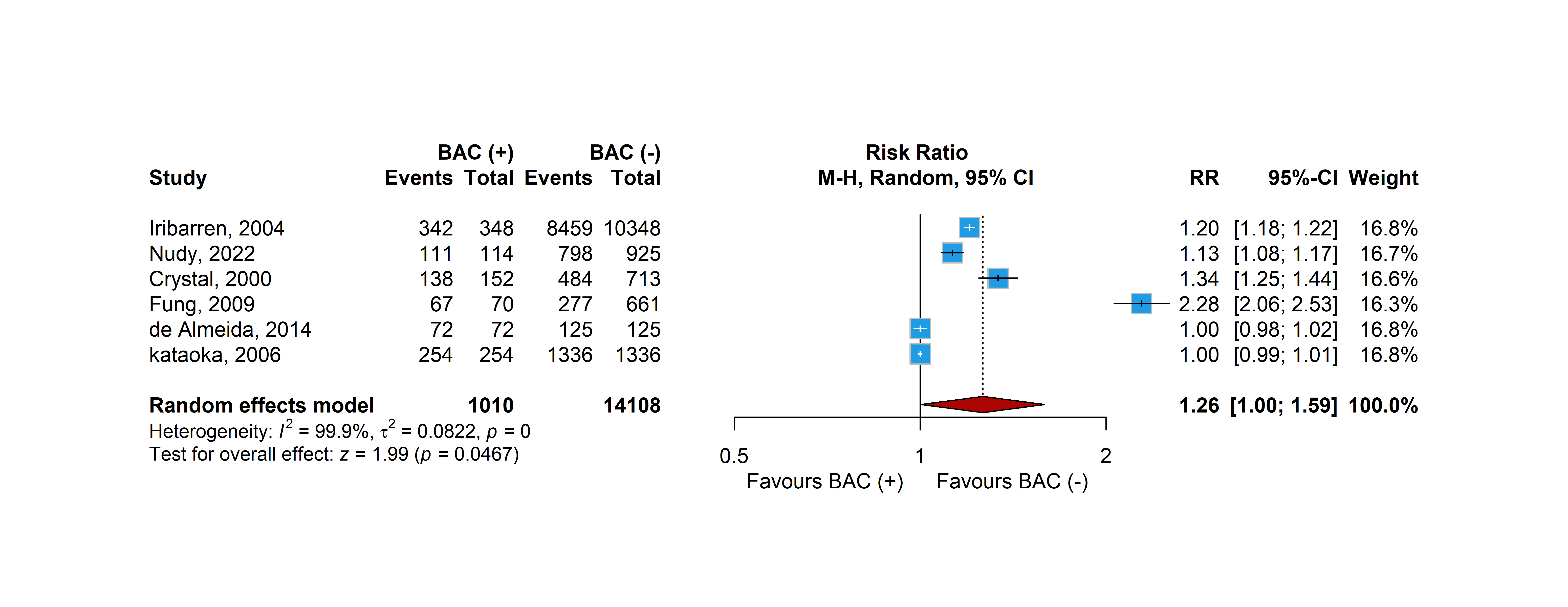


**Figure S2. Pooled risk ratio for current smoking in BAC-positive versus BAC-negative women.**


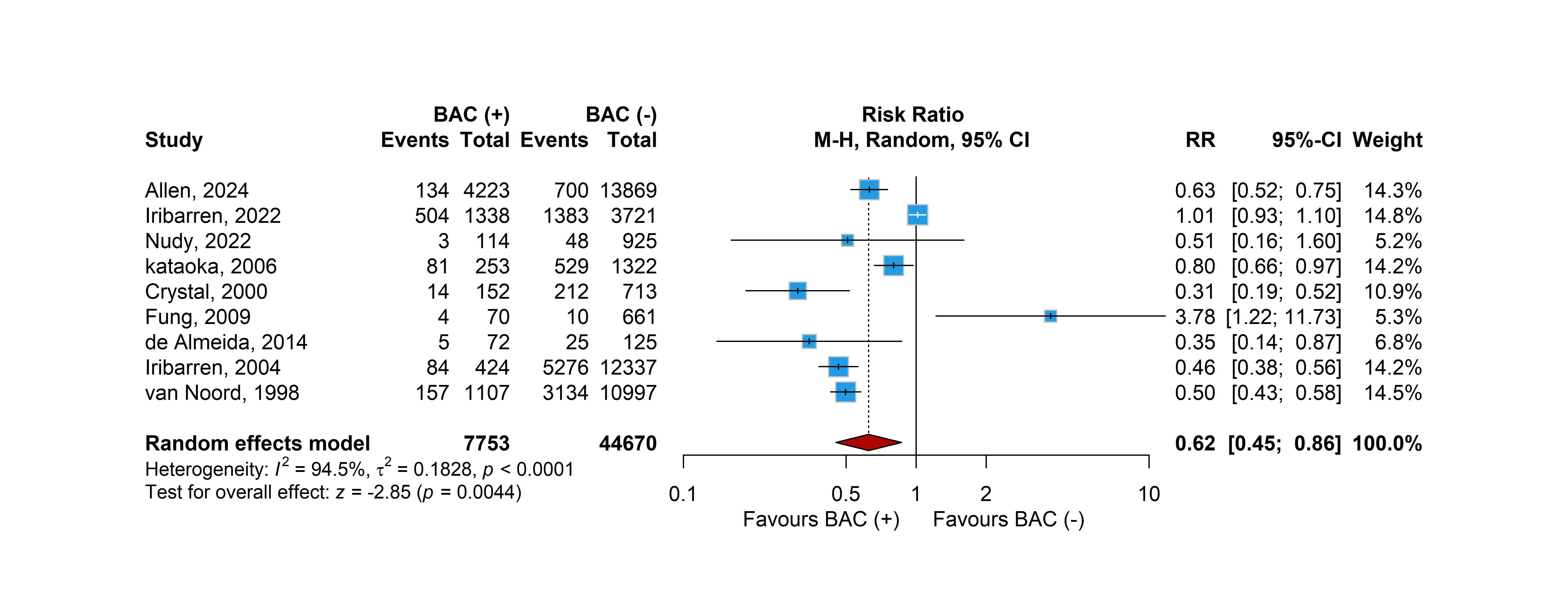


**Figure S3. Pooled risk ratio for use of antihypertensive in BAC-positive versus BAC-negative women.**


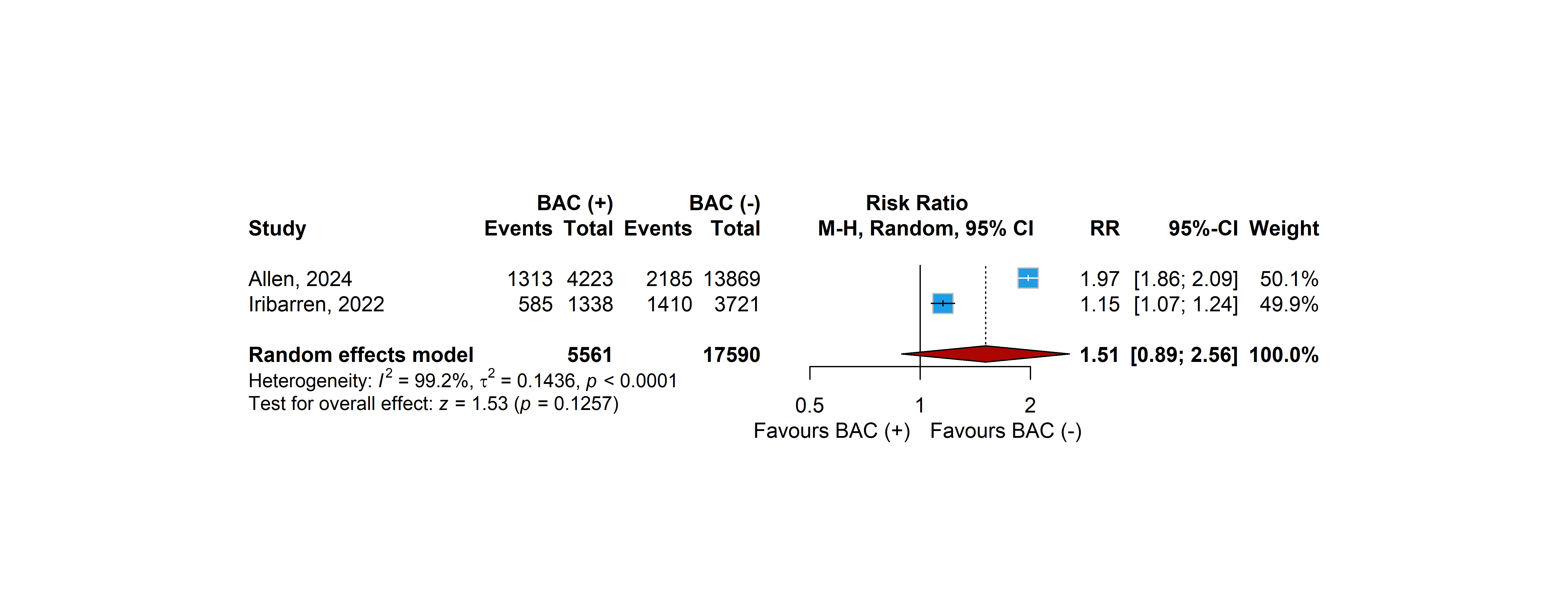


**Figure S4. Pooled risk ratio for lipid-lowering medications (statins) in BAC-positive versus BAC-negative women.**


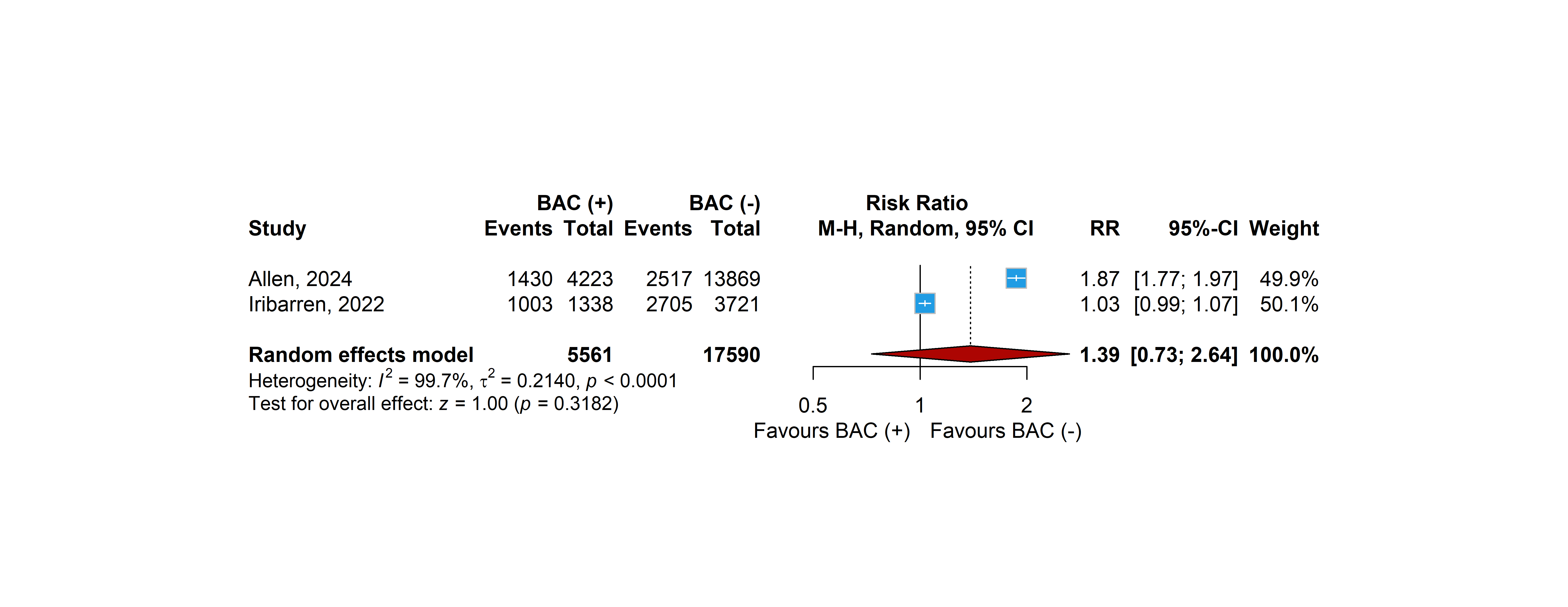


**Figure S5. Pooled risk ratio for total cholesterol levels in BAC-positive versus BAC-negative women.**


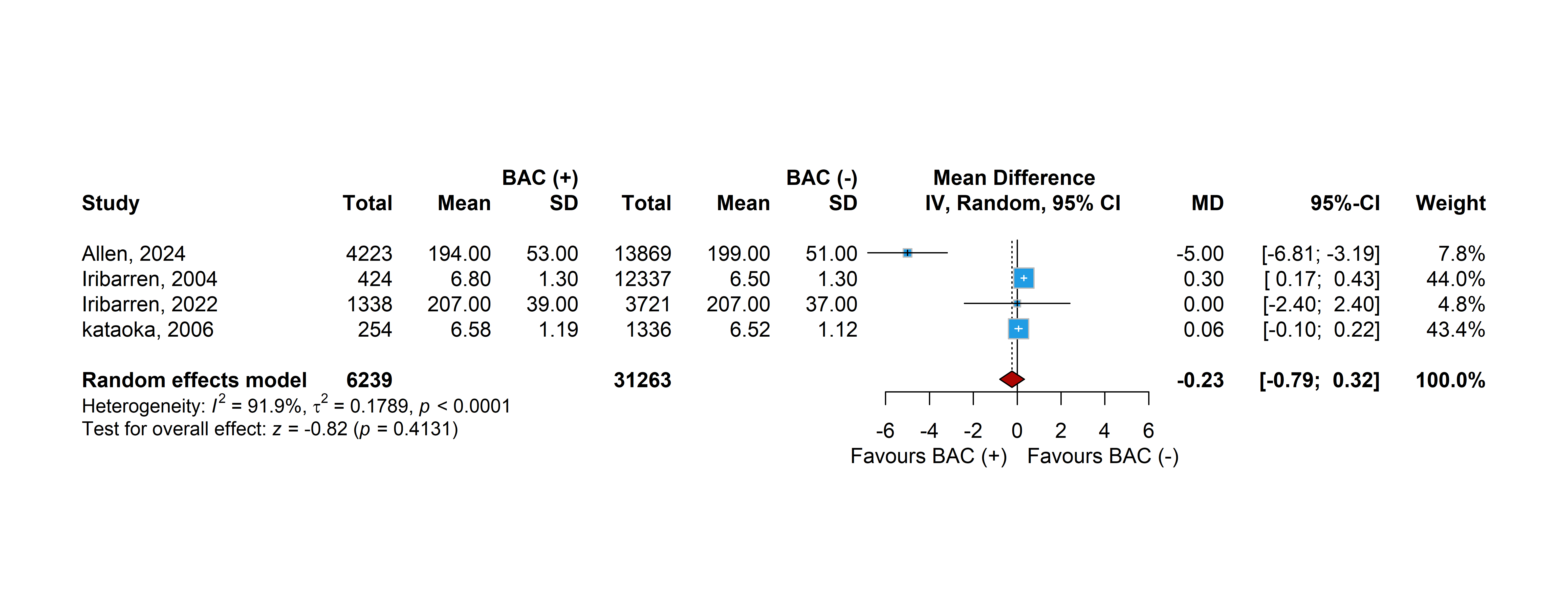


**Figure S6. Pooled risk ratio for history of cardiovascular disease in BAC-positive versus BAC-negative women.**


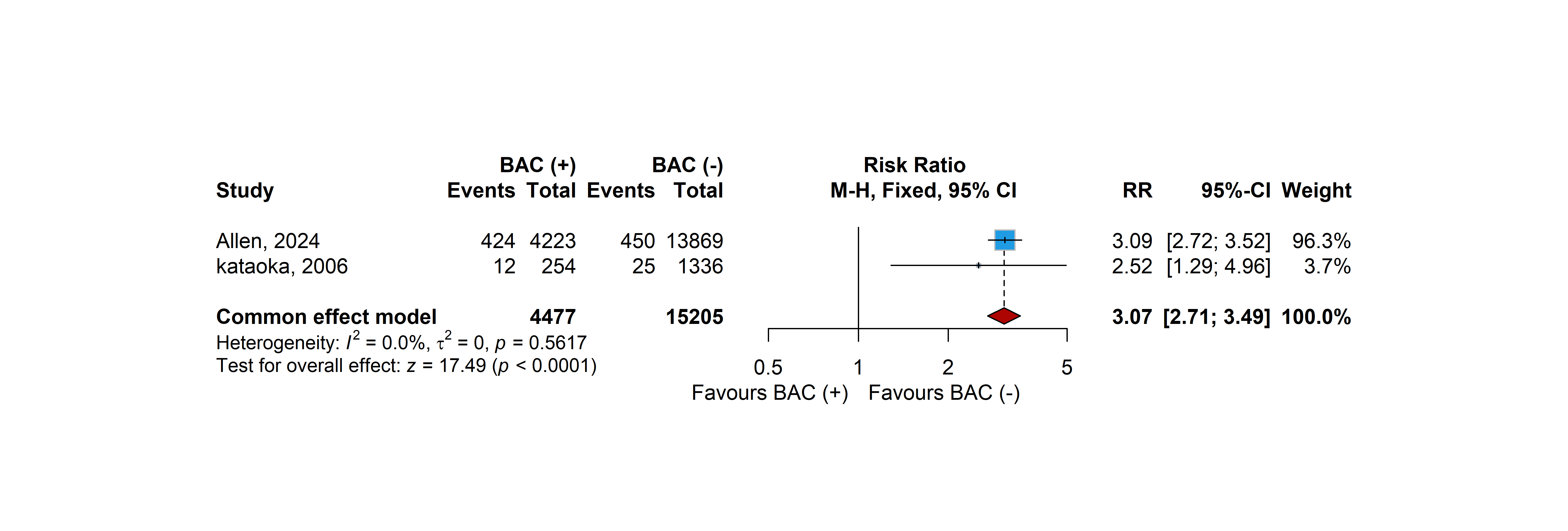


**Figure S7. Pooled risk ratio for current use of hormone therapy in BAC-positive versus BAC-negative women.**


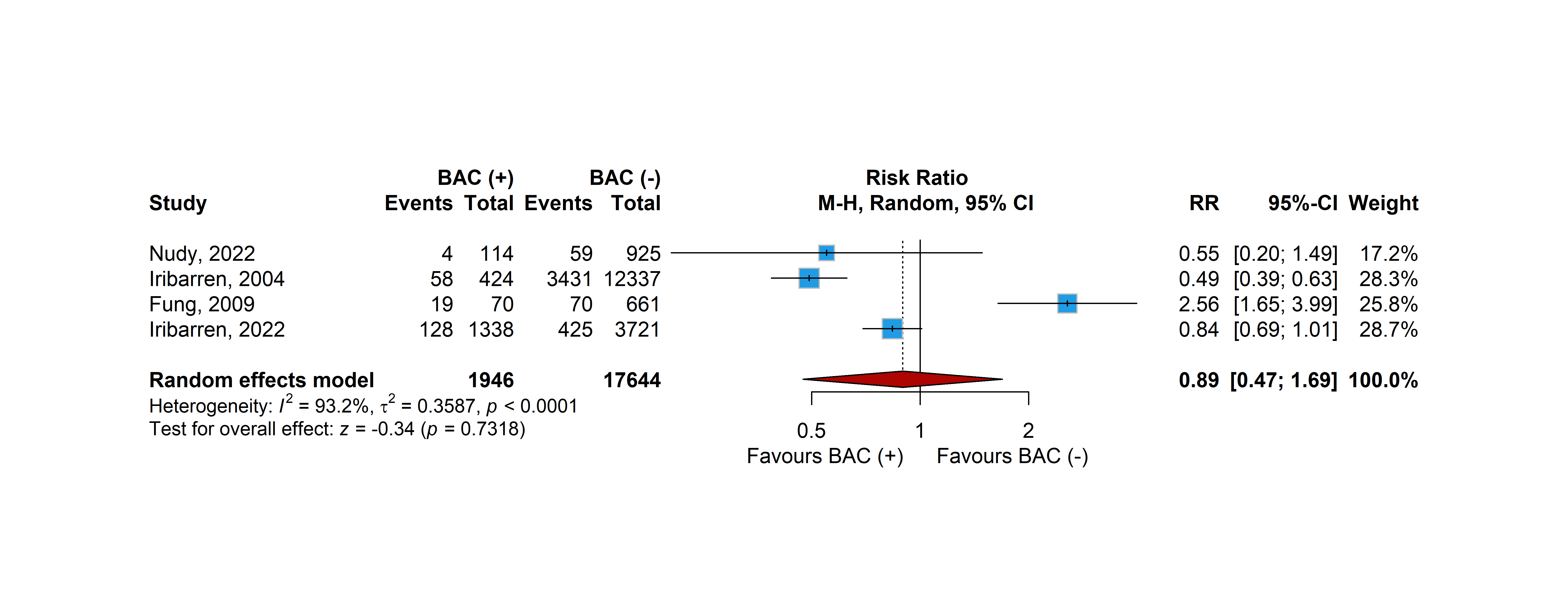


**Figure S8. Funnel plot for publication bias.**

**
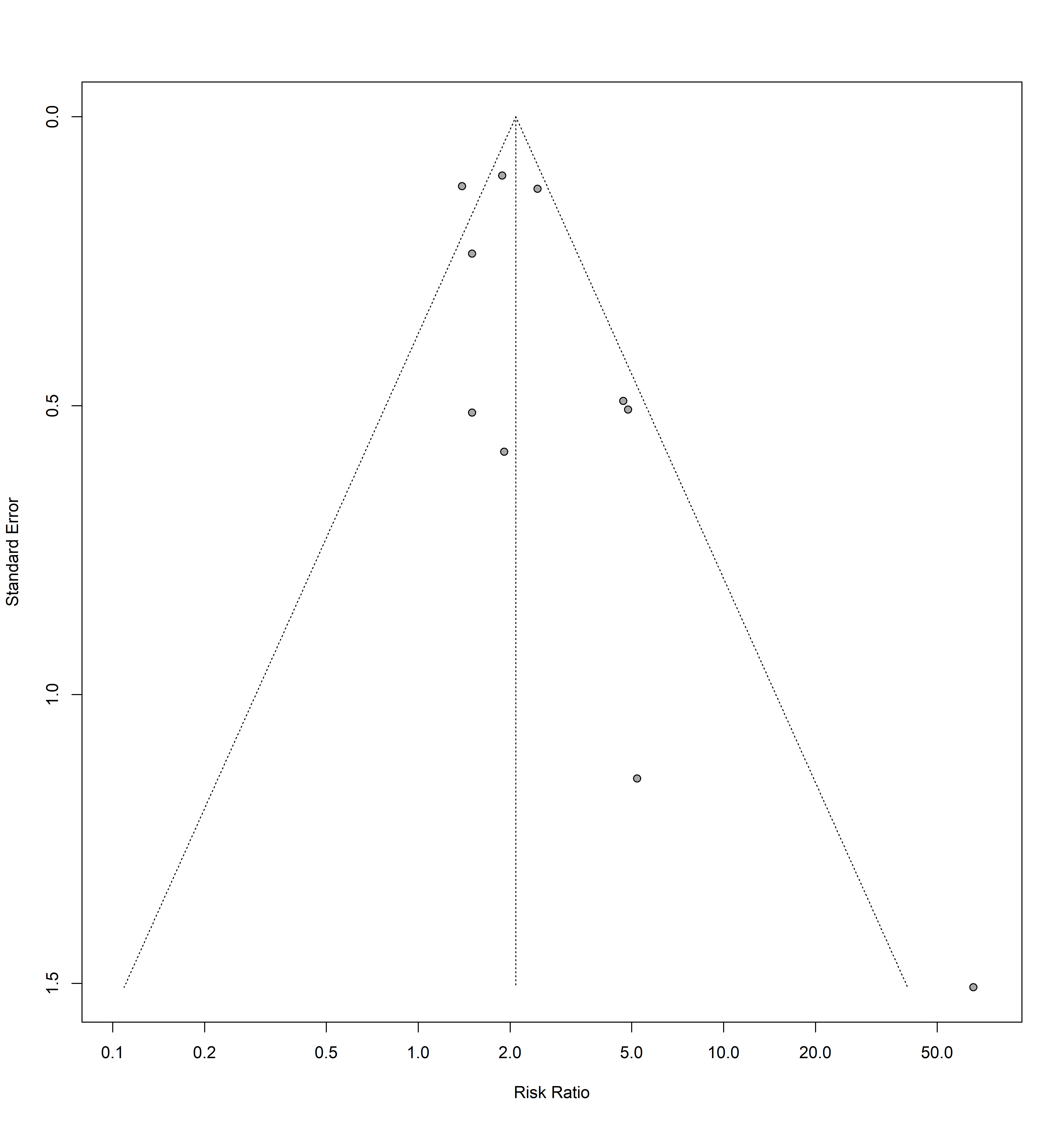
**

**Figure S9. Leave-one-out forest plot - influence of individual studies.**

**
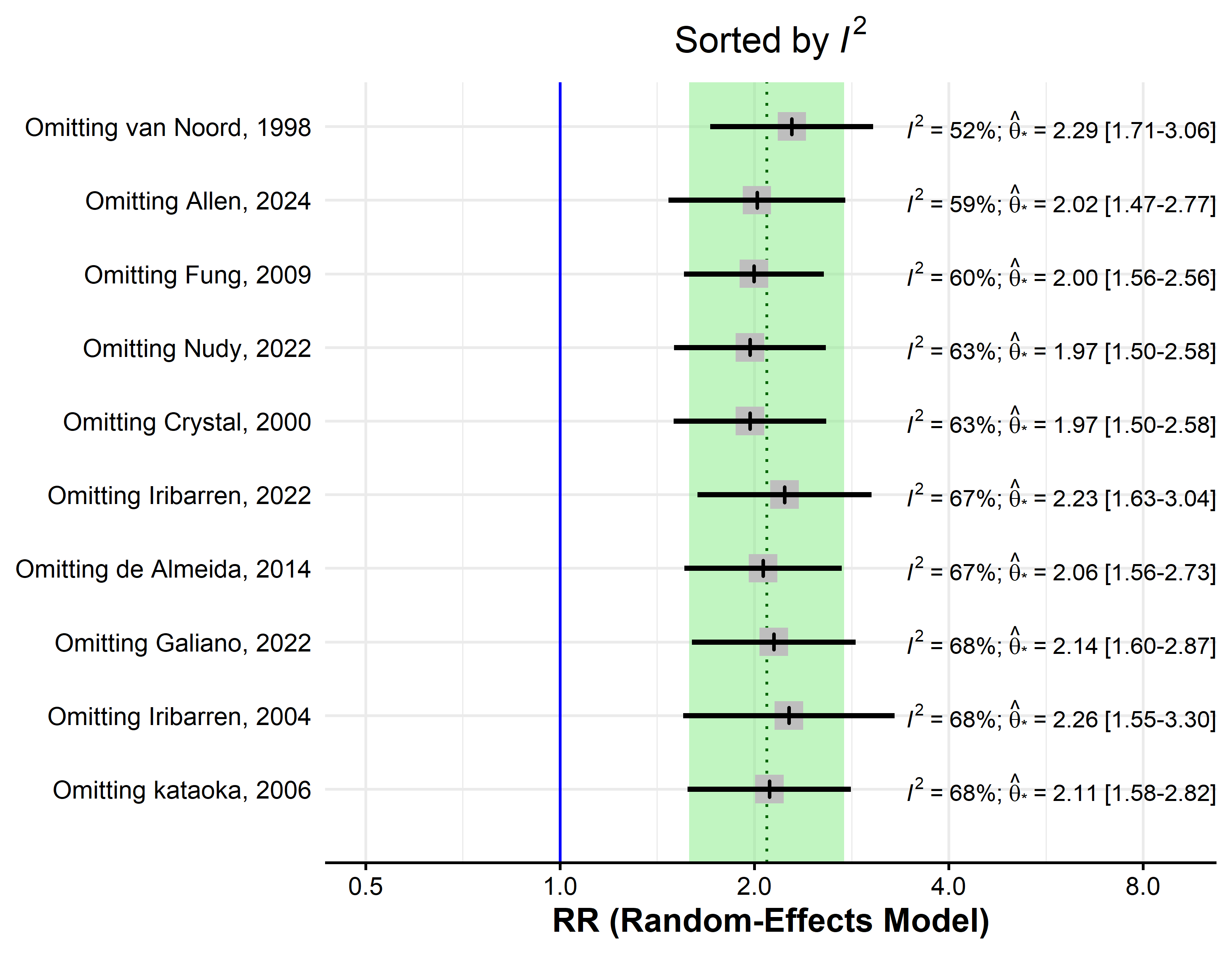
**

**Figure S10. Influence diagnostics plots**

**
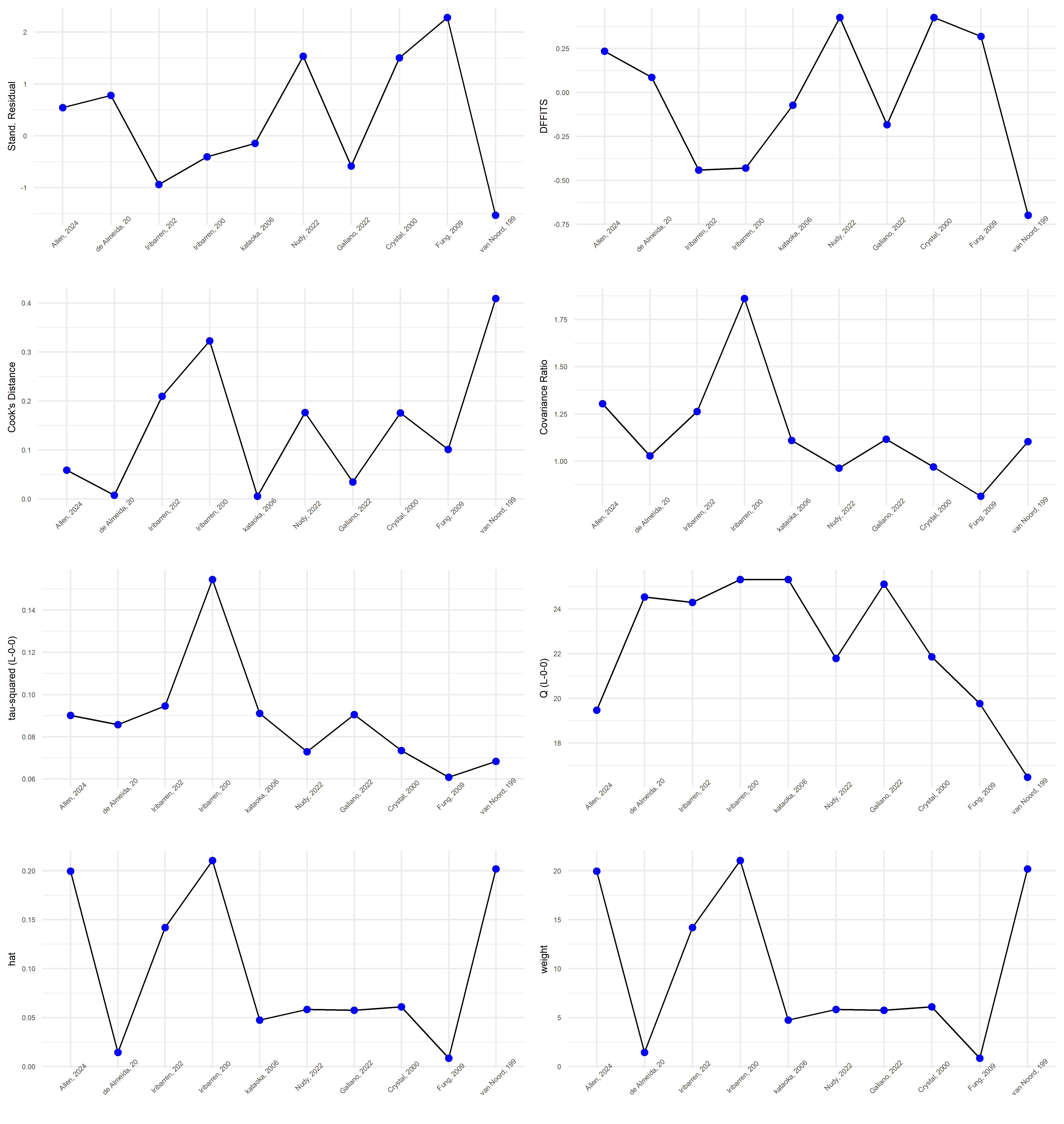
**

**Figure S11.** **Distribution of responses across appraisal domains using the Joanna Briggs Institute (JBI) Critical Appraisal Checklist for Cohort Studies.**


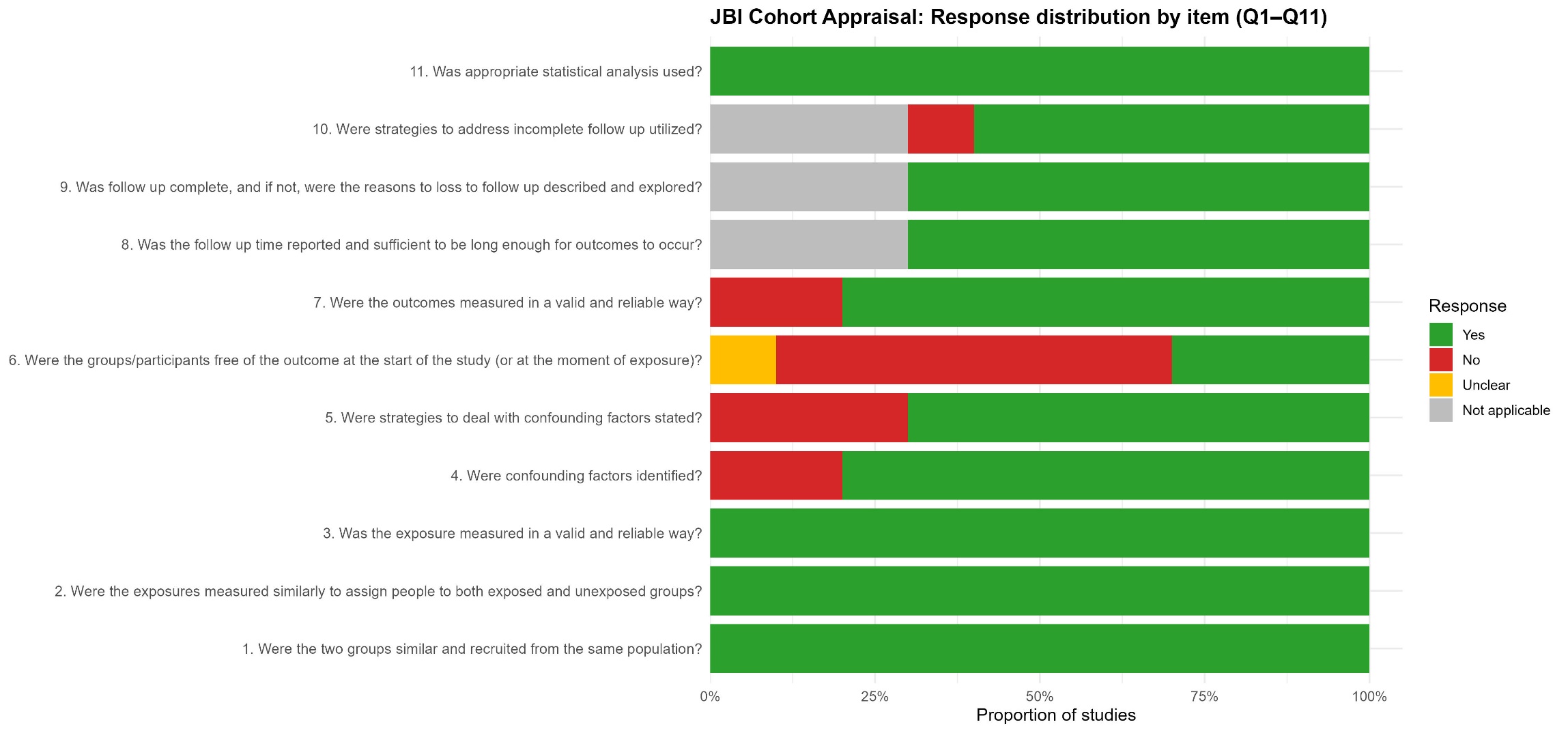


**Figure S12.Traffic-light plot of per-study methodological appraisal using the JBI Critical Appraisal Checklist for Cohort Studies.**


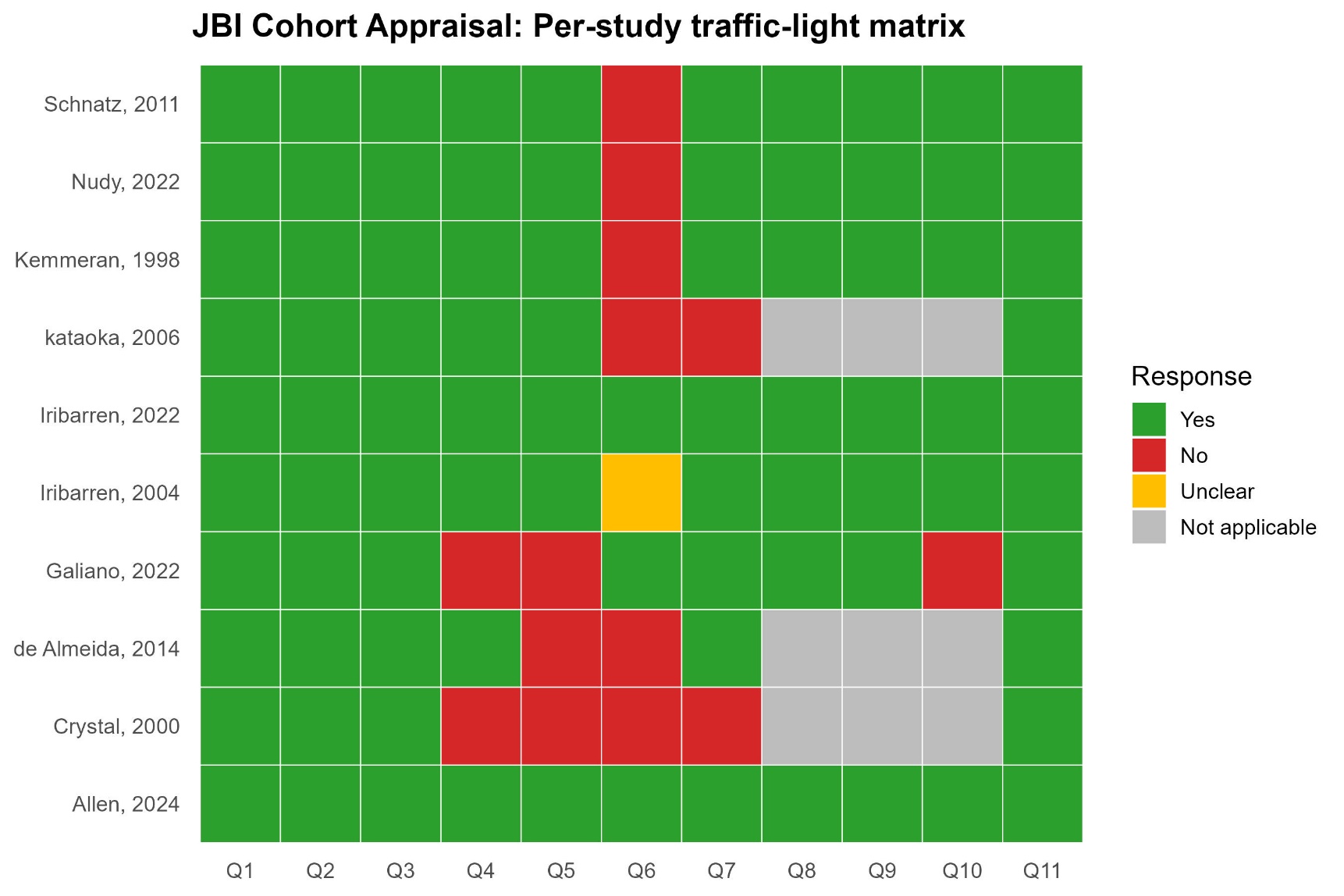

Supplement: Supplementary file 1 [file Table_1.DOCX]
